# Present-Day Problems

**Published:** 1888-12-01

**Authors:** 


### The Doctor's Pulpit.

### PRESENT-DAY PROBLEMS.

(*Continued from page* 115.)

### "At hand-grips with destiny."

Readers of "Sartor Resartus" will remember the phrase which heads this paper. Carlyle seemed to think it was a terrible thing to be in threatened straits for want of the bare means of life. He did not appear to realise, or he chose to forget, that nine out of every ten of living persons are always in these straits. The population of the world as a whole is in daily and deadly conflict with the powers that make for poverty, starvation, and death. The hold of human creatures upon physical life is of the very feeblest. Suppose a man to be alone in Sahara. Let him be chained to a stone and left without food and drink. In four days, if no one comes to his relief, he will be dead, and the vultures will be feeding on him. The few are provided for against all contingencies; the many, the whole world, practically, live from hand to mouth.

Three things are necessary for bare life: food, clothing, lodging. This is at any rate true in our own climate. For healthy life, as distinguished from mere existence, the food must be sufficient and wholesome, the clothing must be suitable, and the lodging healthy and comfortable. Several other conditions are more or less necessary to life on the highest health-level. But the three named are indispensable to any kind of life. Without them, life is not on the whole worth living. Those who are not born to such conditions and who cannot continue in them when they reach the age of self-dependence, had better not have been born at all.

It is not reasonable or valorous to complain of the fact that the vast majority of mankind are always "at hand-grips with destiny." If the present writer reads the finger-posts of life aright, man was intended by Providence to live in such a condition. The order of Nature provides for daily supplies during the summer-time of vegetable growth, and for periods of a few months during the wintry and inactive season. But Nature knows nothing of stocks and shares, or even of banking accounts. Before the invention of coined money, hoarding of any kind was exceedingly difficult, and was a process the limits of which were soon reached. Hoarding for generations and for centuries, as is done now by a highly-artificial civilisation, would have been considered not only a thing impracticable, but as undesirable as it was impossible.

But though there is no just ground of complaint in the fact that Nature intends every man to strip for a bold wrestle with Destiny, there is much reason for dissatisfaction if the chances are ten to one in favour of Destiny and against the man. Supposing Destiny to assume the gaunt and terrible form of Hunger, and to be accompanied by her twin sisters Nakedness and Homelessness; then, indeed, it is not only desirable but imperative that he who has to come to hand-grips with her should have all the courage, all the resolution, all the physical and mental energy and skill of which the typical man is capable. Are we in this country, and at the present time, rearing a race the majority of whom possess these characteristics? We are not.

On a well-ordered farm it is the duty of some to see what fields are to be ploughed, what are to be sown, what crops are to be weeded, and what are to be reaped. It is quite as clearly the duty of others to see that the ploughs, and hoes, and scythes, and other tools, but above all, that the working horses are in a fit condition for their work. For if the horses are by any means rendered incapable, it does not matter though the fields are there, the seed there, the crop ripe in a distant field, and all the tools lying ready. The horses that do the work are indispensable. It is precisely thus in the state. Whilst some must make laws, impose taxes, declare war, or treat for peace, it is the duty of others to see that the men and women who are to obey the laws, to pay the taxes, to fight the battles, or to till the fields in time of peace, are in a condition of sound and vigorous health to do all these things that are imperatively required of them. Can a broken-down cab horse draw a brewer's dray laden with full beer casks? No more can broken-down men and women fight battles, earn money to pay taxes, or preserve a conscience which will impel them to choose obedience to the laws under which they live. It is the duty of somebody to see to the condition of the working animals, if the farm of the state is to be well-cultivated and to pay its way. On whom does this duty naturally devolve? On those who are trained to understand the question—on doctors and on physiologists.

Doctors and physiologists, it is admitted, have neglected the duty which the evolution of civilisation has plainly marked out for them. Certain excuses may be made for their past neglect. For example, it may be said that doctors two or three hundred years ago were not doctors at all, as we now understand the term. They were more or less pretenders. It is a fact which makes us, their descendants, blush, though we are in no sense responsible for it. Physiologists too may claim that theirs is essentially a modern science, that it is but of yesterday; and that they have not had time to consider it in all its evident bearings on the state and its progress, as well as on the individual. These excuses furnish only a partial justification for the slowness of doctors in understanding the full significance of their own art and science from a public point of view. They constitute hardly any excuse at all for their want of enthusiasm in spreading abroad a knowledge of the facts and principles of physiology. Such knowledge is as necessary to the full and perfect life of the body as a reasonable religion is to the life of the soul. If the leaders and pioneers of medical and physiological science be compared with the leaders and pioneers of new religious faiths—of Christianity, for example—how cold, how narrow, how fleshless and bloodless, how pedantic, how inconsequent, how unworthy many of them appear! It is true there are some exceptions to this rule. Huxley, for example, has spared no pains to make his science of physiology thoroughly "understanded of the people." His books may be found, not only in colleges and at public schools, but in Board schools, at mechanics' institutes, at working men's clubs, and, side by side with the "Pilgrim's Progress" and "Uncle Tom's Cabin," on the bookshelves of cottages in every town and village.

But the dawn of a better day, we may hope, is at hand. The seeds of physiological and medical science have not fallen entirely among thorns. Some have been scattered on good ground, and will bring forth fruit. Medical men have not yet quite thrown off the mysterious encasements in which their fathers were accustomed to hide themselves and their ignorance. The doctor of the present day does not yet see that mystery was the necessary defence of his ignorant and not always honest professional ancestor. Still less does he perceive that that which was a necessary defence in times of ignorance is a prison, an impediment, and an injury in times of knowledge. The public, seeing the mask, suspect that the face behind it is concealed for a sinister purpose. Nothing of the kind. At the present day medicine has absolutely nothing whatever to conceal. It is as open, as plain, and as honest as the face of the midnight moon.

To those sciences and arts which devote themselves to the health and development of man must the appeal be finally made when considering the progress or decadence of the community. That there is progress or decadence is certain. That the progress or decadence is almost entirely the result of environment is also certain. That environment in the case of human beings is not an incomprehensible and overwhelming mystery, but a set of more or less modifiable circumstances and conditions, is quite as certain as either of the previous propositions. What is wanted is knowledge—knowledge and purpose. For a strong, high-spirited and capable race like the British, at the very summit of scientific knowledge and practical mechanical skill—for us to sit down with folded hands and see our people drift slowly but surely and inevitably down to physiological ruin and national decay—this is the very madness of apathy and incapacity. We must open our eyes to see the forces at work around us. Every specialist, be his specialism what it may, must contribute his quota to the available stock of knowledge and practical skill. The doctor and the physiologist, as they have taken a prominent lead in the work of iconoclasm and the removal of worn-out theories and beliefs, so now they must show that they can build up as well as pull down, can sow and plant as well as root out weeds, can give to the people a gospel of health and life as well as take away a fetich of superstition, ignorance, and death. Our people as a people are "At actual hand-grips with destiny itself." It is for every one of us to brace himself for the conflict, and to resolve that in this, as in other well-fought fields, victory shall be ours.

*(To be continued.)*

